# Mitochondria-Plasma Membrane Contact Sites: Emerging Regulators of Mitochondrial Form and Function

**DOI:** 10.1177/25152564251332141

**Published:** 2025-04-13

**Authors:** Jason C Casler, Matilde V Neto, Thomas Burgoyne, Laura L Lackner

**Affiliations:** 1 Department of Molecular Biosciences, 3270 Northwestern University, Evanston, IL, USA; 2 UCL Institute of Ophthalmology, 4919 University College London, London, UK

**Keywords:** interorganelle (inter-organelle), mitochondrion (mitochondria), plasma membrane, cell biology, electron microscopy, membrane contact sites (MCSs)‌

## Abstract

Sites of close apposition between organelles, known as membrane contact sites (MCSs), are critical regulators of organelle function. Mitochondria form elaborate reticular networks that perform essential metabolic and signaling functions. Many mitochondrial functions are regulated by MCSs formed between mitochondria and other organelles. In this review, we aim to bring attention to an understudied, but physiologically important, MCS between mitochondria and the plasma membrane (PM). We first describe the molecular mechanism of mitochondria-PM tethering in budding yeast and discuss its role in regulating multiple biological processes, including mitochondrial dynamics and lipid metabolism. Next, we discuss the evidence for mitochondria-PM tethering in higher eukaryotes, with a specific emphasis on mitochondria-PM contacts in retinal cells, and speculate on their functions. Finally, we discuss unanswered questions to guide future research into the function of mitochondria-PM contact sites.

## The Mitochondria-PM Contact Site

**Figure 1.**
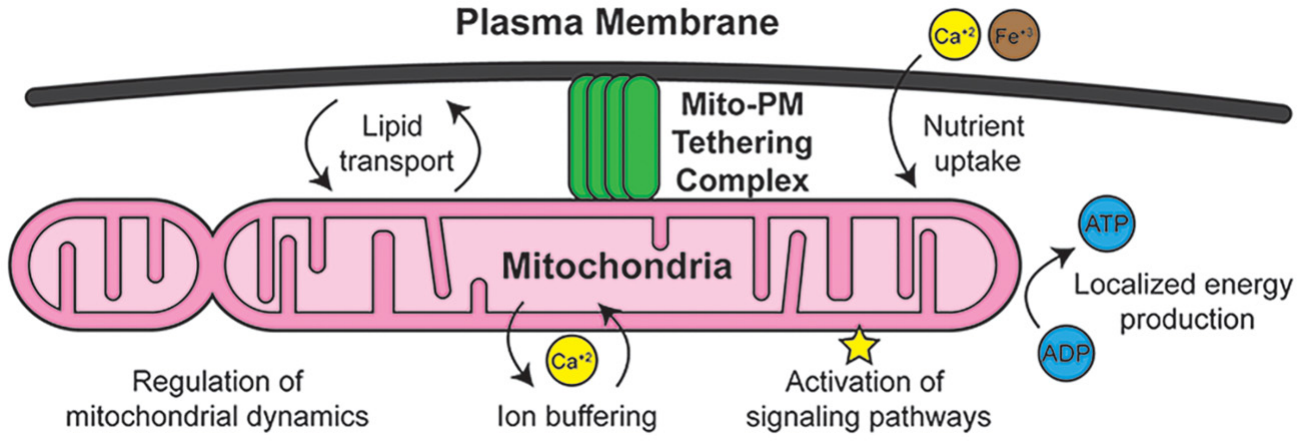
Mitochondria-PM contact sites. A cartoon giving an overview of the structure and role of mitochondria-PM contact sites.

Mitochondria perform dozens of metabolic and signaling functions that are adapted to the physiological requirements of specific cell types (Monzel et al., 2023). To perform their myriad tasks, mitochondria are highly regulated and are constantly being dynamically remodeled. Sites of close apposition between mitochondria and other organelles, known as membrane contact sites (MCSs), have emerged as key regulators of mitochondrial function (Scorrano et al., 2019). MCSs between mitochondria and some organelles, such as the endoplasmic reticulum (ER), have been the subject of extensive study (Prinz et al., 2020; Sassano et al., 2022). It is clear, however, that mitochondria contact most, if not all, other organelles, and several of these MCSs are severely understudied (Lackner, 2019). In this review, we will highlight an understudied MCS between mitochondria and the plasma membrane (PM). Mitochondria-PM contact sites are conserved throughout eukaryotes and are particularly prevalent in cells that experience high bioenergetic demands, such as cardiomyocytes and neurons (Figure 1; Montes de Oca Balderas, 2021). As such, mitochondria-PM contact sites may play significant, unappreciated roles in human health and disease.

## Mitochondria-PM Contact Sites in Yeast

Yeast are the only organisms where mitochondria-PM tethers have been characterized at the molecular level. In the first section of this review, we will describe the molecular mechanism by which yeast tether mitochondria to the PM, the known functions of mitochondria-PM contact sites, and the conservation of mitochondria-PM contacts amongst yeasts.

### Discovery of a Mitochondria-PM Tethering complex in *S. cerevisiae*

**Figure 2.**
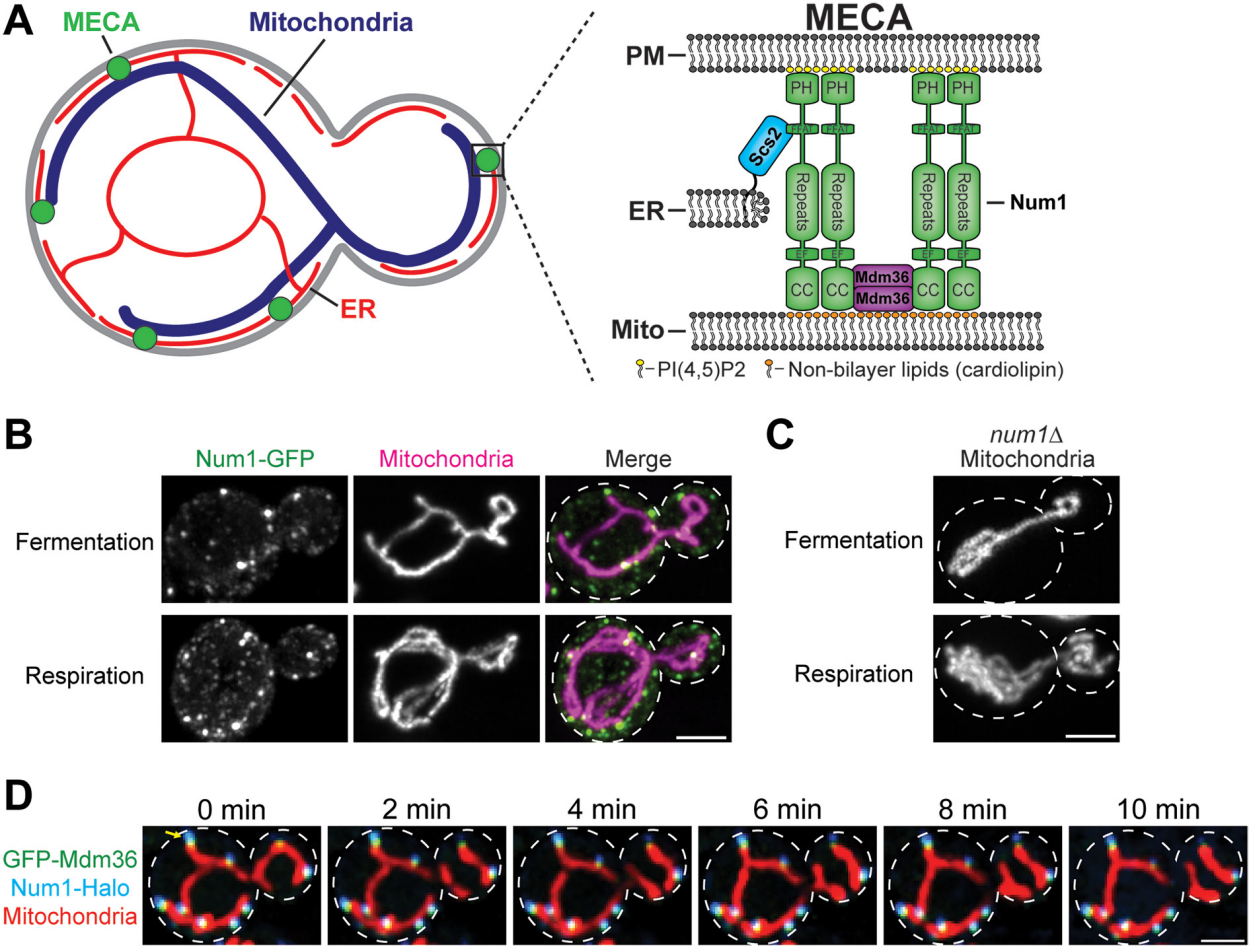
The mitochondria-ER-cortex anchor. (A) A cartoon representation of the intracellular distribution and protein components of MECA. MECA forms cortical foci that tether mitochondria to the ER and PM. The precise stoichiometry of the protein components is unknown. (B) MECA is upregulated during respiratory growth. The images are fluorescence micrographs of the MECA component Num1 tagged with GFP and a mitochondrial matrix marker, MitoRed. Cells were grown in fermentation (glucose media) or respiration (glycerol media) conditions. White dashed lines indicate cell outlines. Scale bar 2 µm. (C) Loss of MECA alters the distribution of the mitochondrial network. Images are fluorescence micrographs of a mitochondrial matrix marker in *num1*Δ cells grown in fermentation (glucose media) or respiration (glycerol media) conditions. White dashed lines indicate cell outlines. Scale bar 2 µm. (D) MECA tethers mitochondria to the PM. Images are fluorescence micrographs of a movie of cells expressing GFP-Mdm36, Num1-Halo, and a mitochondrial matrix marker. The yellow arrow highlights a striking example where a MECA focus tethers the tip of a mitochondrial tubule to the cell cortex over the ten-minute time course. White dashed lines indicate cell outlines. Scale bar 2 µm. All fluorescence micrographs in this figure are from *S. cerevisiae* cells grown and imaged using methods described in Casler et al. (2024).

The first mitochondria-PM tether was discovered in the budding yeast *S. cerevisiae* (Figure 2A). Num1, the central protein component of the yeast mitochondria-PM tether, was identified as a regulator of mitochondrial positioning in a genetic screen to find suppressors of a dominant mutant of the mitochondrial division protein Dnm1 (Cerveny et al., 2007). In this study, it was first reported that cells lacking Num1 lose the cortical distribution of their mitochondrial networks (Figure 2B and C). An additional genetic screen and subsequent functional studies identified a role for Mdm36 in regulating mitochondrial morphology, potentially through promoting an interaction between Num1 and Dnm1 (Dimmer et al., 2002; Hammermeister, 2010). Finally, immunoprecipitation-mass spectrometry and live cell imaging experiments revealed that Num1 and Mdm36 form a complex that physically tethers mitochondria to the PM (Klecker et al., 2013; Lackner et al., 2013). Unexpectedly, this study also identified ER membranes at Num1-mediated mitochondria-PM contact sites. The tripartite MCS was dubbed the mitochondria-ER-cortex anchor, or MECA (Figure 2A; Lackner et al., 2013).

### The Molecular Mechanism of Mitochondria-ER-PM Tethering by MECA

MECA consists of three known protein components—Num1, Mdm36, and Scs2—and tethers mitochondria to the ER and PM (Figure 2A; Casler et al., 2024; Lackner et al., 2013). Num1 is a multifunctional protein that regulates several biological processes. Outside of its role as a mitochondria-PM tether, Num1 was first identified as a cortical dynein anchor that helps position the mitotic spindle during cell division (Farkasovsky and Küntzel, 1995, 2001; Heil-Chapdelaine et al., 2000; Kormanec et al., 1991). Loss of Num1 has no effect on growth, but results in an accumulation of multinucleated cells due to improper positioning of the mitotic spindle (Farkasovsky and Küntzel, 2001; Kormanec et al., 1991). The mechanisms of dynein anchoring and activation by Num1 have been studied extensively and reviewed elsewhere (Greenberg et al., 2018; Omer et al., 2018; Tang et al., 2012). As such, this review will primarily focus on Num1 as an organelle tether, but will highlight where the organelle tethering and dynein anchoring functions overlap.

Num1 is a large 313 kDa protein that contains an N-terminal coiled-coil (CC) domain, an EF-hand like motif (EFLM), twelve 64 AA repeats, a two phenylalanines in an acidic tract (FFAT) motif, and a C-terminal plekstrin homology (PH) domain (Figure 2A; Casler et al., 2024; Kormanec et al., 1991; Tang et al., 2012; Yu et al., 2004). The EFLM binds calcium and plays a role in regulating dynein anchoring, but it is dispensable for organelle tethering (Anderson et al., 2022). Likewise, the 64 AA repeats do not obviously affect any of Num1's known functions (Lackner et al., 2013; Tang et al., 2012). In contrast, the PH domain, CC domain, and FFAT motif are required for organelle tethering.

Mitochondria-PM tethering is mediated by two distinct lipid-binding domains on opposing termini of Num1 (Figure 2A). The Num1 PH domain has a high affinity for phosphatidylinositol 4,5-bisphosphate (PI(4,5)P_2_) and is both necessary and sufficient to mediate targeting to the PM (Tang et al., 2009, 2012; Yu et al., 2004). Mitochondrial tethering is mediated by a direct interaction between the Num1 CC domain and phospholipids on the mitochondrial outer membrane (Ping et al., 2016; Tang et al., 2012). The Num1 CC domain binds negatively charged, cone-shaped phospholipids with a preference for cardiolipin and phosphatidic acid (Ping et al., 2016). Loss of the CC domain disrupts lipid binding and mitochondria-PM tethering (Lackner et al., 2013; Tang et al., 2012). The presence of the Num1 CC domain is both necessary and sufficient to drive the localization of the protein to mitochondria (Ping et al., 2016). Although the CC and PH domains constitute the minimal components required to tether mitochondria to the PM, the formation of robust mitochondria-PM MCSs requires another subunit of MECA - Mdm36 (Lackner et al., 2013).

Num1 and Mdm36 colocalize at cortical foci that stably tether mitochondria to the PM (Figure 2D; Casler et al., 2024; Klecker et al., 2013; Lackner et al., 2013). In the absence of Mdm36, Num1 localizes diffusely along the cell cortex and mitochondria-PM tethering is reduced (Hammermeister, 2010; Lackner et al., 2013). In the absence of Num1, Mdm36 is primarily cytoplasmic and cortical mitochondrial tethering is absent (Hammermeister, 2010). These data suggest that Mdm36 regulates the oligomerization of Num1. The Num1 CC domain and Mdm36 both exist as dimers and form a membrane-binding complex in vitro (Ping et al., 2016; Won et al., 2021). While no structural data exist for Num1 or Mdm36, AlphaFold3 predicts a direct interaction between the Num1 CC domain and Mdm36 (Abramson et al., 2024). In vivo photobleaching studies indicate that MECA foci contain ∼30–40 molecules of Num1 (Omer et al., 2021). Deletion or overexpression of Mdm36 drastically reduces or enhances the size of Num1 foci respectively, suggesting that Mdm36 is a limiting factor in determining the size of mitochondria-PM tethering points (Casler et al., 2024; Omer et al., 2021). Thus, Mdm36 may function to concentrate Num1 in an arrangement that supports mitochondrial tethering.

ER tethering is mediated by an interaction between the integral ER protein Scs2, a homolog of the mammalian VAP proteins, and the Num1 FFAT motif (Casler et al., 2024; Chao et al., 2014; Omer et al., 2018). Scs2 recruits FFAT motif-containing proteins to the ER and has been implicated in regulating the metabolism of several different lipid species (Murphy and Levine, 2016). In addition, Scs2 and its paralog Scs22 function as molecular tethers mediating ER-PM contact sites (Manford et al., 2012; Quon et al., 2018). Surprisingly, the organelle tethering functions of Num1 are separable. Mutation of the Num1 FFAT motif or loss of Scs2 prevents the association of Num1 with ER membranes but has no effect on the formation of mitochondria-PM tethering points (Casler et al., 2024; Chao et al., 2014; Omer et al., 2018). Thus, the ER is not an obligate component of mitochondria-PM contact sites, but it does contribute to Num1 functions.

While the molecular components are now well-characterized, MECA has primarily been visualized by fluorescence microscopy and the ultrastructure of this tripartite MCS is unknown. One study observed PM invaginations that contact the mitochondrial outer membrane via electron tomography (Klecker et al., 2013). Further work was unable to clarify the precise nature of these structures and whether these PM invaginations represent common mitochondria-PM contact sites remains unknown, however (Klecker et al., 2013; Westermann, 2015). Analysis of MECA via electron microscopy has likely been hampered by the relatively low number of contact sites per cell in nutrient rich growth conditions. Future studies using conditions that enhance MECA formation, such as growth in respiratory conditions or the overexpression of Mdm36, coupled with advanced electron microscopy techniques, such as correlative light and electron microscopy, may be required to characterize the ultrastructure of mitochondria-PM contact sites in yeast.

### MECA Functions

MECA organizes a cortical hub that facilitates communication between three separate organelles. In this section, we will summarize what is known about MECA's various functions and describe how these functions relate to organelle tethering.

#### Promoting Mitochondrial Function

The biological significance of positioning the mitochondrial network proximal to the cell cortex is not entirely clear (Montes de Oca Balderas, 2021). Mitochondria carry out complex biochemical reactions that require specific metabolites, such as iron and calcium, whose intracellular concentrations are often low and tightly controlled (Monzel et al., 2023). Therefore, an attractive hypothesis is that mitochondria near the PM have greater access to nutrients that are transported into the cell. In support of this hypothesis, yeast lacking Num1 have a decreased oxygen consumption rate, which is indicative of mitochondrial dysfunction (White et al., 2022). Additionally, during respiratory growth, MECA is upregulated and the amount of mitochondria tethered to the cell cortex increases (Figure 2B and C; White et al., 2022). These changes suggest that increasing mitochondria-PM contact sites promotes mitochondrial function during times of high bioenergetic demand. An important next step will be to determine whether mitochondria positioned near the PM have distinct biochemical profiles and how this contributes to their function.

MECA may also influence mitochondrial function by regulating other mitochondrial MCSs. Recently, it was demonstrated that the distribution and biogenesis of the ER-mitochondria encounter structure (ERMES), a mitochondria-ER MCS that is involved in lipid transport, is positively regulated by mitochondria-PM tethering (Casler et al., 2025; Kornmann et al., 2009). Surprisingly, synthetic mitochondria-PM tethering is sufficient to restore ERMES distribution in the absence of MECA. This study provides direct evidence that mitochondria-PM tethers are involved in regulating the spatial organization and dynamics of mitochondrial MCSs, which likely contributes to promoting mitochondrial function (Casler et al., 2025).

#### Promoting Mitochondrial Division

Num1 was originally proposed to be required for mitochondrial division (Cerveny et al., 2007). However, rather than the hyperfused mitochondrial nets seen in mitochondrial division machinery mutants, loss of Num1 results in a disorganized mitochondrial network with multiple tubules overlapping one another in the center of the cell (Figure 2B and C; Bleazard et al., 1999; Klecker et al., 2013; Lackner et al., 2013). In addition, loss of Num1 only results in a ∼50% reduction in the rate of mitochondrial division, rather than a complete loss (Harper et al., 2023; Lackner et al., 2013). These observations suggest that Num1 positively influences mitochondrial division, rather than being strictly required for it to occur.

**Figure 3.**
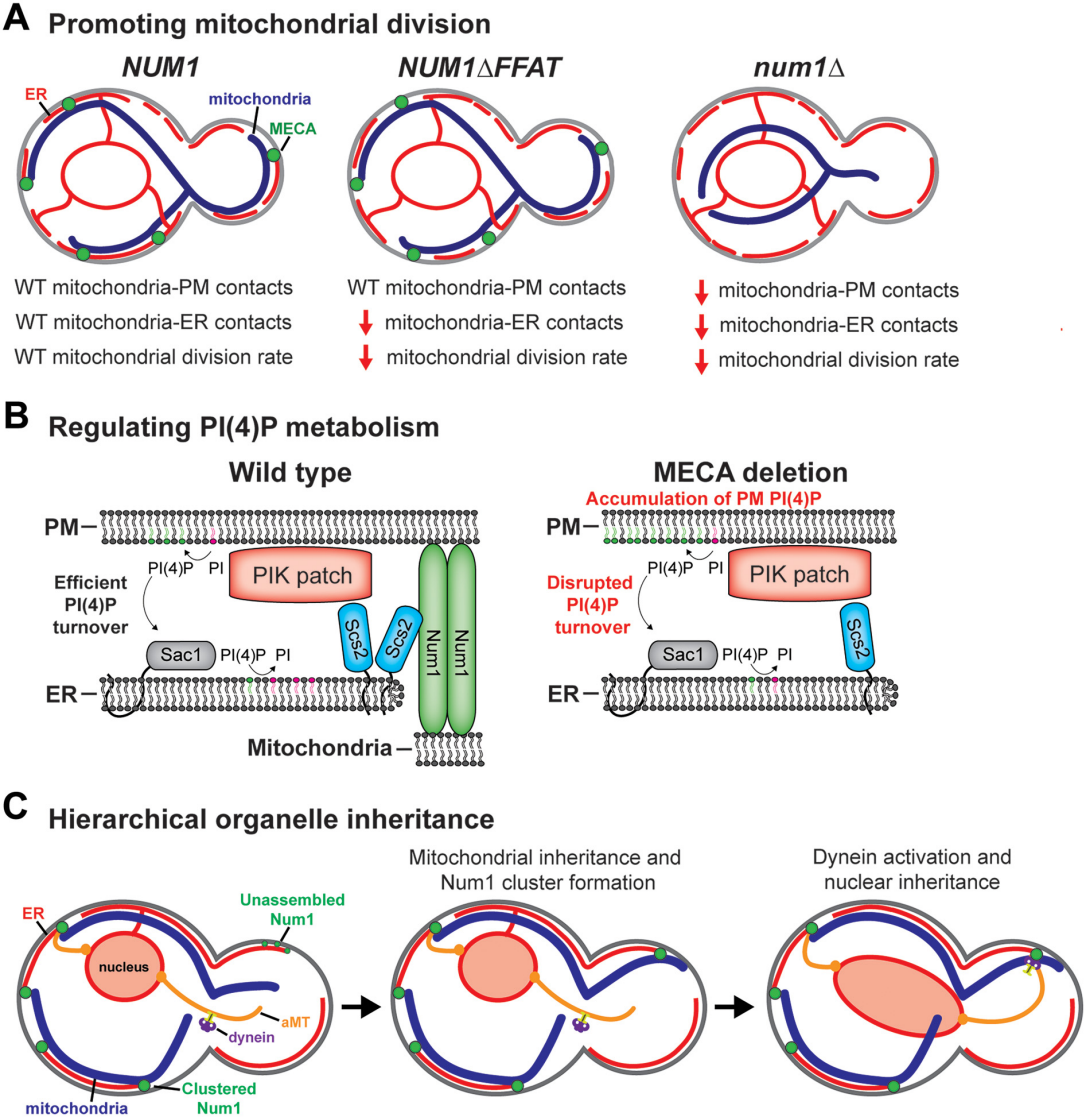
Functions of MECA. (A) MECA promotes mitochondrial division. Cells expressing full-length Num1 have robust mitochondria-ER-PM tethering and wild-type rates of mitochondrial division. Cells expressing Num1ΔFFAT have reduced mitochondria-ER contacts, wild-type levels of mitochondria-PM contacts, and reduced mitochondrial division rates. Cells lacking Num1 have reduced mitochondria-ER contacts, reduced mitochondria-PM contacts, and reduced mitochondrial division rates. (B) MECA regulates PI(4)P metabolism. A cartoon representation of the interaction between MECA and PIK patches. PIK patches synthesize PI(4)P (green phospholipids) from PI (magenta phospholipids) on the PM. PI(4)P is transported to the ER where it is dephosphorylated by Sac1. In wild-type cells, MECA localizes adjacent to PIK patches and regulates the efficiency of PI(4)P turnover. In MECA deletion mutants, PI(4)P turnover is disrupted and PI(4)P accumulates on the PM. Whether mitochondrial membranes play an active role in regulating PI(4)P metabolism is unknown. (C) MECA influences the hierarchical inheritance of mitochondria prior to nuclear inheritance. As the cell cycle progresses, unassembled pools of Num1 associate with the ER and PM in the growing bud. Next, the inheritance of mitochondria drives the clustering of Num1, resulting in the formation of new stable mitochondria-ER-PM contact sites. Dynein is preferentially anchored and activated at mitochondria-associated Num1 clusters to promote nuclear inheritance.

Several mechanisms have been proposed to explain the role of Num1-mediated mitochondria-PM tethering in regulating mitochondrial division. First, Num1 was suggested to promote mitochondrial division by interacting with the mitochondrial division machinery (Cerveny et al., 2007). Interestingly, a subset of Dnm1 foci colocalize with Num1 on the cell cortex in a manner dependent on the adaptor Caf4 (Casler et al., 2024; Cerveny et al., 2007; Lackner et al., 2013). Loss of Caf4 does not result in a substantial mitochondrial division defect, however, suggesting that the interaction between Num1 and Dnm1 is not required to promote mitochondrial division (Griffin et al., 2005). Next, Num1-mediated mitochondria-PM tethering may generate tension on the mitochondrial membrane (Klecker et al., 2013; Westermann, 2015). In this model, the active transport of mitochondria by the actin-dependent myosin motor Myo2 is counterbalanced by Num1-mediated mitochondria-PM tethering, which generates membrane tension to promote mitochondrial division (Westermann, 2015). In support of this model, expression of a synthetic mitochondria-PM tether partially rescues mitochondrial morphology and mitochondrial division defects in the absence of Num1 (Klecker et al., 2013). In contrast, a separate study saw no change in the rate of mitochondrial division after the induction of a distinct Num1-independent synthetic mitochondria-PM tether (Harper et al., 2023). Neither study directly measured mitochondrial membrane tension, however, and differences in the synthetic tethering systems may account for the contrasting results. Finally, it was most recently suggested that Num1's role as a mitochondria-ER tether rather than a mitochondria-PM tether regulates mitochondrial division. In cells expressing Num1ΔFFAT, an allele of Num1 that tethers mitochondria to the PM but not to the ER, mitochondrial division rates phenocopy a full Num1 deletion (Figure 3A; Casler et al., 2024). This result strongly suggests that mitochondria-ER tethering is the primary mechanism by which Num1 regulates mitochondrial division. In support of this, mitochondria-ER MCSs have been firmly established as regulators of mitochondrial division (Friedman et al., 2011). Importantly, the precise mechanism by which mitochondria-ER contacts, including those formed by Num1, influence mitochondrial division is unknown, and is the subject of intense study (Tábara et al., 2025).

#### Mitochondrial Partitioning

The presence or absence of mitochondria-PM tethering is important for mitochondrial partitioning during mitotic and meiotic cell division, respectively. In mitotically growing cells, MECA anchors mitochondria to the cortex of the mother cell and new MECA foci form in the bud at a late stage of the cell cycle (Klecker et al., 2013; Kraft and Lackner, 2017). The polarized distribution of MECA contributes to the selective maintenance of mitochondria in the mother cell (Chelius et al., 2023; Klecker et al., 2013). Num1 and Mmr1, a protein involved in mitochondrial inheritance, show opposite effects on the distribution of the mitochondrial network (Chelius et al., 2023; Klecker et al., 2013). Loss of Num1 increases the amount of mitochondria transported to the daughter cell while loss of Mmr1 increases the amount of mitochondria retained in the mother cell (Klecker et al., 2013). The function of these proteins in mitochondrial partitioning has been speculated to play a role in regulating mitochondrial quality control during stress or aging (Chelius et al., 2023).

In contrast to the remarkable stability of MECA in mitotically dividing cells, MECA is degraded during meiosis. As meiosis progresses, Num1 and Mdm36 are phosphorylated and subjected to proteasomal degradation to promote mitochondrial detachment from the mother cell cortex (Sawyer et al., 2019). Detached mitochondria then associate with the nascent gamete nuclei. One speculation is that degradation of MECA contributes to the selective inheritance of specific populations of mitochondria (e.g., healthier mitochondria) by the newly formed gametes via an unknown mechanism (Sawyer et al., 2019). Taken together, these results demonstrate that the presence or absence of mitochondria-PM tethering can be used to control the spatial distribution of the mitochondrial network for different purposes.

#### PI(4)P Homeostasis

Recently, it was demonstrated that MECA interacts with phosphoinositide kinase (PIK) patches (Casler et al., 2024). PIK patches are PM-localized protein complexes that catalyze the synthesis of phosphatidylinositol-4-phosphate (PI(4)P) from the precursor phosphatidylinositol (Baird et al., 2008). Loss of Num1 or any of its organelle tethering capabilities alters the distribution of PI(4)P on the PM, likely by disrupting PI(4)P turnover at ER-PM contact sites (Figure 3B; Casler et al., 2024). PI(4)P is involved in regulating polarized secretion and recruiting proteins to the PM (Balla, 2013; Hammond et al., 2012). Thus, mitochondria-PM contacts may have a previously unappreciated role in regulating PI(4)P-dependent processes. Interestingly, connections between PI(4)P metabolism and the regulation of mitochondrial division have now been reported in mammalian tissue culture, *Drosophila*, and yeast (Casler et al., 2024; Enkler et al., 2023; Nagashima et al., 2020; Terriente-Felix et al., 2020). An exciting avenue of exploration will be to dissect the functional connections between mitochondria-PM contacts, PI(4)P metabolism, and mitochondrial dynamics in other model systems. While these are seemingly disparate cell biological activities, one underlying connection is they are all regulated by Scs2/VAPs. Loss of Scs2/VAPs results in disrupted PI(4)P metabolism at ER-PM contact sites and reduced rates of mitochondrial division in both yeast and mammalian cells (Boutry and Kim, 2021; Casler et al., 2024; Hammond et al., 2012; Stefan et al., 2011). Thus, one interesting line of investigation will be to decipher how competition for binding to the same ER protein, Scs2/VAP, may influence the coordinated regulation of these processes. Finally, beyond PI(4)P, it will be important to examine the impact mitochondria-PM contacts have on the composition and organization of other proteins and lipids in the PM and, consequently, on PM functions.

#### Hierarchical Organelle Inheritance

Num1 functions in both the mitochondrial and nuclear inheritance pathways. As the cell cycle progresses, newly formed clusters of Num1 anchor mitochondria and dynein to the cell cortex in the growing bud (Kraft and Lackner, 2017). Dynein anchoring and activation help orient the mitotic spindle to facilitate nuclear inheritance (Markus and Lee, 2011). Interestingly, the mitochondria and dynein tethering functions of Num1 are linked. Mitochondrial inheritance is required for new Num1 clusters to form in the bud, and, unexpectedly, disrupting mitochondrial inheritance results in spindle orientation defects (Kraft and Lackner, 2017). Studies using synthetic Num1 clustering systems further demonstrated that dynein is preferentially offloaded onto mitochondria-associated Num1 clusters via a mechanism that requires calcium binding by the Num1 EFLM (Anderson et al., 2022; Schmit et al., 2018). Remarkably, in the evolutionarily distant *Schizosaccharomyces pombe,* dynein is also preferentially anchored at mitochondria-PM tethering sites (Kraft and Lackner, 2019). Importantly, mitochondria are not strictly required for Num1 to bind and activate dynein, as non-mitochondria associated Num1 can also function in nuclear inheritance (Omer et al., 2021). Despite this, strongly biasing dynein anchoring to mitochondria-associated Num1 clusters may contribute to the fidelity of organelle inheritance by ensuring nuclear inheritance occurs after mitochondrial inheritance (Figure 3C; Anderson et al., 2022). Thus, mitochondria-PM contacts play a role in the fidelity of organelle inheritance in budding yeast.

### Mitochondria-PM Tethering in Other Yeasts and Unicellular Eukaryotes

**Figure 4.**
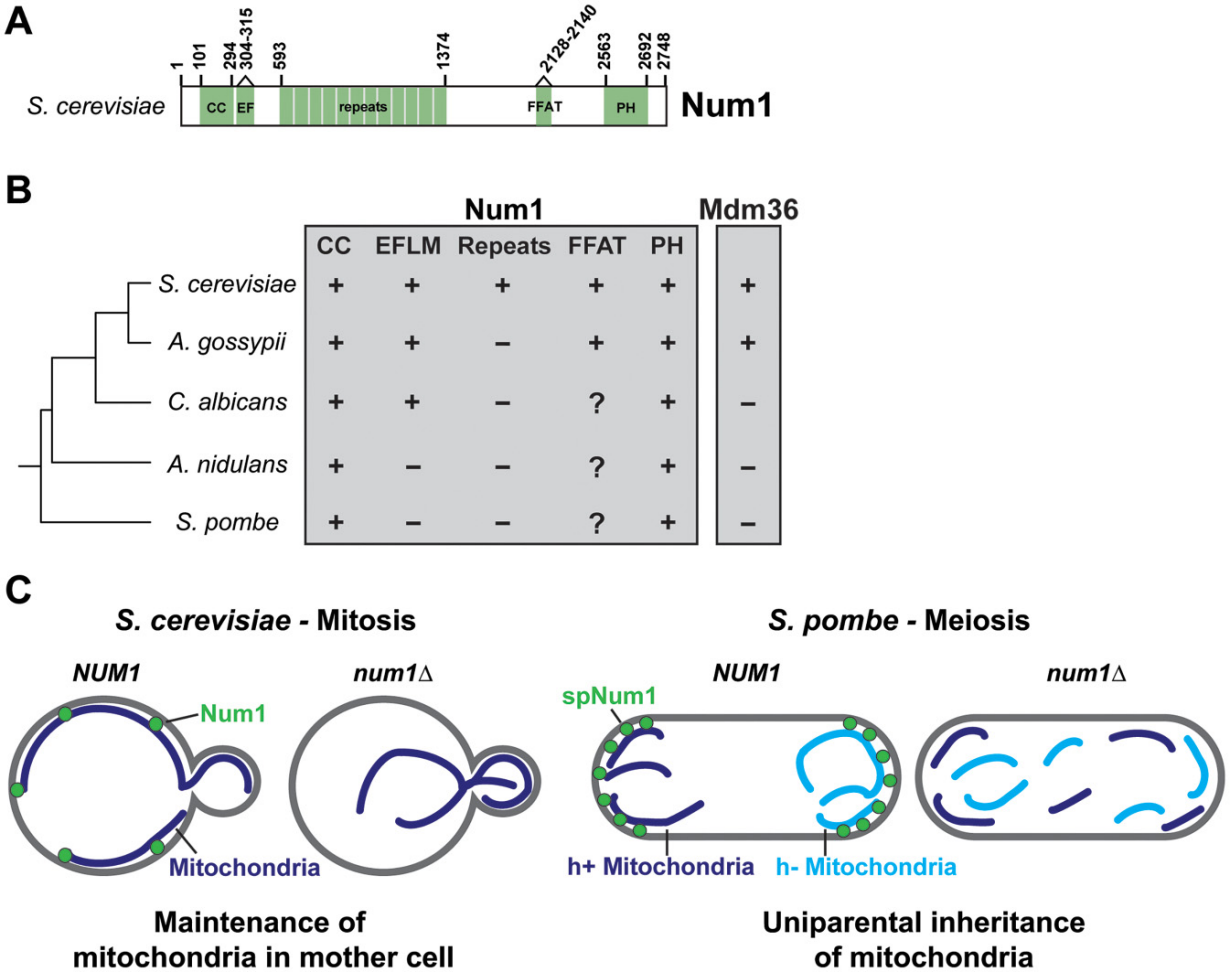
Conservation of mitochondria-PM contact sites in yeasts. (A) A cartoon representing domains and motifs present in *S. cerevisiae* Num1. (B) A comparison of the conservation of Num1 and Mdm36 amongst yeast. Conservation of Num1 is further broken down into discrete domain structures. A “+” indicates presence, a “−” indicates absence, and a “?” indicates an unknown. The CC and PH domains are highly conserved and annotated for each yeast species on Uniprot (The UniProt Consortium 2023). The presence of a putative EFLM was determined by analyzing potential calcium-binding residues in the N-termini of Num1 homologs via IonCom (Hu et al., 2016). “Repeats” refers specifically to large (>50) AA repeats, similar to the 64 AA repeats in *S. cerevisiae* Num1. FFAT motifs are difficult to predict due to a large tolerance of variation from the canonical FFAT motif sequence (Slee and Levine, 2019). A putative FFAT motif was identified in *A. Gossypii* Num1 (IISSSSEYTDALD), however, due to significant similarity in sequence and location to the experimentally validated *S. cerevisiae* FFAT motif (STTTSSMFTDALD). The *A. nidulans* section is specifically in reference to the Num1 homolog ApsA. The presence of putative Mdm36 homologs was predicted using OrthoDB (Kuznetsov et al., 2023). (C) In *S. cerevisiae*, mitochondria-PM tethering mediated by Num1 aids in the retention of mitochondria in the mother cell during mitotic division. Loss of Num1 results in an increase in the amount of mitochondria transported to the growing bud. In *S. pombe*, expression of spNum1 during meiosis facilitates the selective retention of mitochondria from a single parent in a subset of gametes. The absence of spNum1 results in the mixing of parental mitochondrial populations during meiosis.

Num1 is highly conserved amongst fungi, but its role in mitochondrial tethering has been largely unexplored outside of *S. cerevisiae*. The minimal domain architecture that is required for mitochondria-PM tethering, a CC and PH domain, is well conserved amongst Num1 homologs (Figure 4A and B). It seems unlikely, however, that the precise architecture of mitochondria-PM contact sites observed in *S. cerevisiae* is conserved. First, Mdm36 is only expressed in a subset of *Ascomycota* fungi (Figure 4B; Kuznetsov et al., 2023). While Mdm36 is not strictly required for the formation of mitochondria-PM tethering points, it enhances their robustness (Lackner et al., 2013; Ping et al., 2016). Therefore, it is possible that mitochondria-PM tethering may be transient or less stable in other yeasts. Second, the FFAT motif is not obviously conserved, suggesting that the interaction with Scs2 and the formation of tripartite mitochondria-ER-PM contact sites may not occur in all yeasts. The EFLM and repeat regions are also poorly conserved (Kuznetsov et al., 2023). Third, the precise mechanism by which the Num1 CC domain binds phospholipids is unknown, making it difficult to predict which Num1 homologs will interact with mitochondrial membranes. Finally, some fungi express multiple Num1 paralogs (Kuznetsov et al., 2023). Despite this, the high degree of conservation of Num1 highlights a need for comparative studies between different yeast species to identify which features of mitochondria-PM tethering are conserved.

Some important studies towards this goal have already been performed in the fission yeast *S. pombe*. SpNum1, also known as Mcp5, contains an N-terminal CC domain and a C-terminal PH domain that share 36% and 48% similarity, respectively, to their *S. cerevisiae* counterparts (Kraft and Lackner, 2019). The CC domain of SpNum1 binds mitochondrial phospholipids and expression of SpNum1 in *S. cerevisiae* is sufficient to tether mitochondria to the PM (Kraft and Lackner, 2019). Thus, despite an estimated evolutionary distance of over 450 million years, the molecular mechanism of mitochondria-PM tethering is conserved between *S. pombe* and *S. cerevisiae*. There are several interesting differences between Num1 and SpNum1, however. In contrast to Num1 which is expressed in mitotically growing cells and degraded during meiosis, SpNum1 is only expressed during meiosis (Chacko et al., 2019; Kraft and Lackner, 2019; Saito et al., 2006; Sawyer et al., 2019). Despite the difference in expression, mitochondria-PM tethering may play a similar role in both systems. During meiotic division in *S. pombe*, expression of SpNum1 is required for the uniparental inheritance of mitochondria (Figure 4C; Chacko et al., 2019). Clusters of SpNum1 physically tether mitochondria from each parent to the PM at opposite cell poles. Similarly, Num1-mediated mitochondria-PM tethering aids in the retention of mitochondria in the mother cell during mitotic cell division (Klecker et al., 2013). Thus, expression of either Num1 or SpNum1 supports the physical segregation of populations of mitochondria within a continuous cytoplasm (Figure 4C). A likely possibility is that mitochondria-PM tethering is finely tuned to facilitate the different requirements of each species.

Outside of yeast, the role of mitochondrial tethering to the cell periphery has also been studied in the single-cell eukaryotic parasite *Toxoplasma gondii*. During the lytic cycle, the mitochondrial networks of *T. gondii* residing within a host cell show a strong positional bias to the cell periphery which is lost upon transition to the extracellular matrix (Ovciarikova et al., 2017). The changes in mitochondrial positioning are reversible upon return to the host, strongly implying an active tethering mechanism maintains the peripheral distribution (Ovciarikova et al., 2017). While the precise functions of mitochondria-PM contact sites in *T. gondii* are unknown, the observation that they form in a regulated manner suggests biological significance. Therefore, further work studying mitochondria-PM contact sites may yield crucial insights into the regulation of host-parasite interactions.

## Mitochondria-PM Contact Sites in Mammalian Cells

In contrast to yeast, there are few reports of mitochondria-PM contacts in mammalian systems. The distribution of the mitochondrial network in higher eukaryotes varies considerably between different cell types, and strong subplasmalemmal localization is not always observed. Furthermore, the tethering proteins identified in yeast, Num1 and Mdm36, do not have mammalian homologs, suggesting divergent evolutionary paths for these contact sites (Montes de Oca Balderas, 2021). Despite the few studies showing mitochondria-PM tethers, multiple studies have found mitochondria in close proximity to the PM and some functional relevance has been proposed for these interactions.

### Functional Interactions Between the Mitochondria and the PM

#### Regulation of Calcium Fluxes in the PM by Subplasmalemmal Mitochondria

Mitochondria play a pivotal role in regulating calcium signaling at the PM, with significant implications for cellular physiology and pathology. Of particular interest are the subplasmalemmal mitochondria, which form a specialized microdomain near the PM that can modulate calcium ion diffusion (Demaurex et al., 2009; Frieden et al., 2005). These organelles swiftly sequester calcium, creating localized low-calcium microenvironments that influence the activity of calcium-dependent ion channels and transporters in the PM. One example of this was shown in mouse pancreatic acinar cells (Park et al., 2001). In these cells, the peripheral basolateral mitochondria uptake Ca^2+^, leading to an increase in ATP production that supports Ca^2+^-activated ATPases in the ER. By sequestering Ca^2+^ into both the ER and mitochondria, the cellular system reduces local Ca^2+^ concentration near the entry sites. This reduction helps mitigate the strong negative feedback effect that would otherwise interrupt Ca^2+^ channel activity, thereby maintaining the store-operated Ca^2+^ entry mechanism. These findings were corroborated by a subsequent study, where the authors showed that in the absence of mitochondria, there was a disruption in the transfer of Ca^2+^ from the PM channels to the ER (Frieden et al., 2005). The consequent increase in the subplasmalemmal Ca^2+^ concentration leads to the upregulation of the activity of PM Ca^2+^-ATPase (PMCA) pumps. Moreover, the lack of a Ca^2+^ replenishing mechanism provided by mitochondria results in a Ca^2+^ depletion in the ER, further emphasizing the importance of these organelles in subplasmalemmal Ca^2+^ homeostasis.

A recent study unveiled the role of a tripartite MCS formed between the PM, ER, and subplasmalemmal mitochondria in EGF receptor (EGFR) degradation by non-clathrin endocytosis (NCE) (Mesa et al., 2024). Stimulation with high concentrations of EGF was shown to lead to the assembly of the mitochondria-ER-PM contact sites, with mitochondria serving two critical functions. Firstly, they act as calcium buffers, creating Ca^2+^ oscillations that maintain intermittently high Ca^2+^ concentrations near the platform, thereby preventing feedback closure of the inositol 1,4,5-triphosphate receptor (IP3R) ER channel by Ca^2+^. Secondly, the Ca^2+^ oscillations within mitochondria lead to an increase in ATP production, which is essential for several processes, including the formation of tubular invaginations, the fission of EGFR-NCE vesicles, and the induction of collective cell migration. Interestingly, while the currently characterized functions differ substantially, the similar organelle architecture of the tripartite mitochondria-ER-PM contact sites organized by MECA in *S. cerevisiae* and the MCS identified by Mesa et al., in mammalian cells suggests a potentially fundamental biological relevance of tethering these three organelles.

An often-underappreciated aspect of mitochondrial regulation of PM calcium fluxes is the role of ATP. As the primary source of cellular ATP, mitochondria indirectly modulate the function of numerous ATP-dependent or ATP-modulated calcium channels and pumps in the PM. Moreover, high concentrations of ATP have been shown to have a calcium buffering effect in numerous cells, with freely diffusible ATP facilitating the diffusion of calcium (Baylor and Hollingworth, 1998; Eisner et al., 2023; Montalvo et al., 2006).

The intricate relationship between mitochondrial function and calcium homeostasis becomes particularly evident in pathological conditions. Mitochondrial dysfunction can lead to impaired calcium uptake, resulting in elevated cytosolic calcium levels and potential cellular toxicity. Consequent disruptions in ATP production can compromise the function of ATP-dependent calcium channels and pumps, leading to further impairment in calcium signaling. Strikingly, neuronal cells seem to be particularly susceptible to this imbalance in calcium signaling, with various neurodegenerative diseases, such as Alzheimer's, Parkinson's, and Huntington's disease and amyotrophic lateral sclerosis being attributed to disruptions in calcium homeostasis (MacDonald et al., 1993; Martin, 2010; Simon-Gozalbo et al., 2020; Zampese et al., 2011).

#### Immune Cell Migration and Activation

Mitochondria have been shown to be involved in regulating immune cell function. These dynamic organelles serve as critical signaling hubs, influencing immune cell activation, differentiation, and effector functions through metabolic reprogramming, the release of immunogenic molecules, and the modulation of key signaling pathways (Chen et al., 2023; Trinchese et al., 2024). Although no direct contacts between mitochondria and the PM have been clearly demonstrated in immune cells, many important functions of mitochondria in regulating immune responses rely on their close proximity to the PM, from which immune cell migration and activation should be highlighted.

Studies have demonstrated that mitochondria undergo specific translocation and accumulation at the posterior region of migrating immune cells, known as the uropod (Campello et al., 2006). This organelle redistribution close to the PM is facilitated by microtubule-dependent transport mechanisms, involving the interaction between Miro-1, a mitochondrial Rho GTPase, and the dynein motor complex (Morlino et al., 2014). Notably, mitochondrial fission, mediated by proteins such as Drp1, has been shown to enhance this relocation process and subsequently promote lymphocyte chemotaxis (Campello et al., 2006). Although the functional significance of mitochondrial accumulation at the uropod has not yet been unveiled, one possible hypothesis is that this localization serves to provide a concentrated source of ATP, necessary to fuel the energy-intensive processes of actomyosin contraction and uropod retraction (Morlino et al., 2014). This localized energy production is particularly critical in rapidly migrating cells such as lymphocytes, where the demand for ATP is heightened due to their increased motility (Ledderose et al., 2018).

When elucidating the role of mitochondrial positioning in T-lymphocyte activation, studies have revealed a sophisticated mechanism whereby these organelles translocate to the proximity of the PM during immunological synapse formation (Contento et al., 2010). This strategic juxtaposition, of approximately 200 nanometers from the membrane, facilitates the mitochondrial sequestration of calcium ions, thereby preventing the accumulation of high calcium microdomains that could potentially inactivate the crucial ORAI channels. Consequently, this spatial arrangement ensures a sustained calcium influx, optimizing T-cell activation kinetics and amplifying downstream immune responses through enhanced NFAT signaling and subsequent gene transcription (Quintana et al., 2011). In addition to their calcium buffering function, these mitochondria were demonstrated to facilitate localized ATP production, which is crucial for two key processes: the assembly of central supramolecular activation clusters (cSMACs) and the mobilization of T-cell receptor (TCR) micro-clusters. Consequently, mitochondrial activity plays a crucial role in modulating both the intensity and persistence of TCR signaling (Baixauli et al., 2011).

### Mitochondria-PM Tethers in Mammalian Cells

Mitochondria-PM contact sites were first observed in specialized cell-to-cell junctions found in the follicular cells of the thyroid of bats. In this study, the mitochondrial outer membranes were described to run parallel and in close apposition to these junctions of the PMs of adjacent cells (Nunez, 1974). In some instances, both mitochondria and the ER were also shown to colocalize in close apposition to the PM, thus suggesting a potential role of mitochondria in energy provision for intercellular communication and localized synthetic processes.

**Figure 5.**
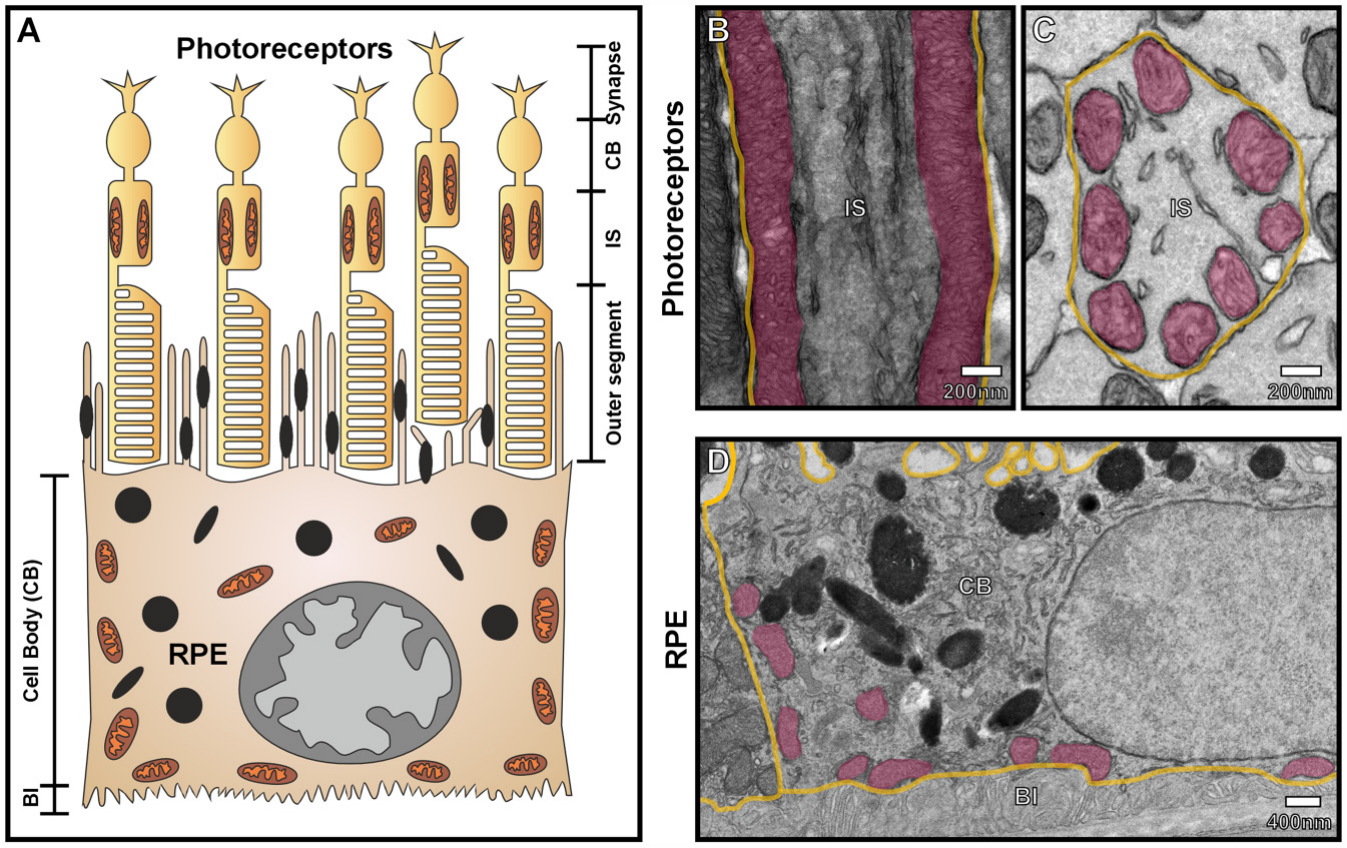
Mitochondria-plasma membrane contact sites in photoreceptors and the retinal pigment epithelium. (A) Diagram showing mitochondria (presented in brown/orange) within the retinal pigment epithelium (RPE) and the inner segment (IS) region of photoreceptors. (B) and (C) Transmission electron microscopy images of photoreceptor IS from a (B) longitudinal and (C) en face view. The mitochondria (purple) are at the periphery of the photoreceptor IS and in contact with the plasma membrane (PM) shown in yellow. (D) Transmission electron microscopy image of the RPE where most mitochondria are in contact with the lateral and basal plasma membrane. The basal plasma membrane forms intricate basal infoldings (BI) and it is these that are in contact with mitochondria. (B–D) Samples were prepared for electron microscopy and imaged using methods described in Meschede et al. (2020) and Neto et al. (2024).

Recently, our ultrastructural studies have shown the presence of mitochondria-PM contact sites in two distinct components of the retina—the photoreceptors and the retinal pigment epithelium (RPE) (Meschede et al., 2020). Mitochondria play a crucial role in the retina due to the exceptionally high metabolic demands to maintain healthy vision. In photoreceptors, mitochondria extend along most of the inner segment region of the cell and are tethered to the PM in a highly organized manner (Figure 5A and B; Meschede et al., 2020). A particularly striking feature of these mitochondria is that, along with their cristae openings, they align with those from neighboring inner segments. This symmetric arrangement potentially serves multiple functions, including energy supply for protein synthesis and phototransduction, calcium buffering, and facilitating the sharing of metabolites between adjacent cells to maintain homeostasis across the photoreceptor layer. Having mitochondria positioned at the PM may also assist in channeling light through the inner segment to focus it onto the light-detecting outer segment region of the cell. The reduction of OPA1, a protein primarily known for its role in the maintenance of mitochondrial homeostasis and cristae remodeling, was demonstrated to alter mitochondria morphology and resulted in their positioning away from the PM. This indicates a new role for this protein in sustaining the specialized mitochondrial arrangement in photoreceptor inner segments.

Within RPE cells, mitochondria have a distinctive ‘carpet-like’ arrangement against the basal surface, which is maintained by tethers that anchor the mitochondrial outer membrane to the PM of the cells (Figure 5; Neto et al., 2024). A possible explanation for this strategic positioning of mitochondria could be to provide efficient delivery of ATP to PM channels and transporters, required for the transport of glucose, ions, and other nutrients across the basal membrane, as well as the removal of metabolic waste products to the highly vascular underlying layer known as the choroid. Mitochondria may also be involved in local calcium signaling and homeostasis. While mitochondria were found to redistribute from the basal to the apical area of the RPE after light onset, the proportion of mitochondria tethered to the PM did not change. The mitochondria-PM contacts were also conserved in a Choroideremia eye disease model, characterized by the abnormal basal membrane morphology and loss of infoldings, which further supports the functional importance of this interaction.

## Conclusion and Future Perspectives

Mitochondria are vital for cellular function and homeostasis, extending far beyond their well-known capacity as the powerhouses of the cell. This includes numerous cellular processes, ranging from ion buffering, lipid metabolism, and immune responses to apoptosis and even aging (Aon et al., 2014; Chen et al., 2023; Giorgi et al., 2018; Glover et al., 2024; Lima et al., 2022; Srivastava, 2017). These dynamic organelles are strategically localized throughout the cytoplasm, which enables efficient energy distribution and allows for rapid communication with multiple organelles, including the ER, Golgi apparatus, lysosomes, and notably, the PM. This review highlights the significant knowledge gap between our understanding of mitochondria-PM contact sites in yeast and mammalian cellular systems. The molecular mechanism and functions of mitochondria-PM contacts are most well defined in budding yeast. Leveraging the strengths of the yeast system, powerful molecular tools and separation of function mutants have been used to dissect how distinct features and activities of the proteins that localize to mitochondria-PM contacts contribute to the formation and function of these sites. In doing so, a growing list of functions, some of which have been quite unexpected, have been attributed to mitochondria-PM contacts in these unicellular eukaryotes. In contrast, while our understanding of mitochondria-PM contact sites in mammals is growing, many questions remain regarding their molecular composition, regulation, and full functional significance. It can be speculated that, due to their increased cellular complexity, mammalian systems are likely to have more intricate regulatory mechanisms. In addition, the functions of mitochondria-PM contacts in mammalian cells are likely to be diverse, reflecting the specialized roles of mitochondria in different cell types and tissues. Thus, further research is needed to characterize mammalian mitochondria-PM contact sites and their potential implications for health and disease.

## Outstanding Questions

1. What are the molecules that mediate mitochondria-PM tethering in higher eukaryotes?
2. What factors regulate the formation of mitochondria-PM contact sites?
3. How do mitochondria-PM contact sites influence mitochondrial function? Are mitochondria near the PM biochemically distinct from mitochondria within the cytoplasm?
4. How are plasma membrane activities, such as endocytosis, exocytosis, and membrane transport, regulated by mitochondria-PM contact sites?
5. How is the spatial organization of the PM, its protein and lipid components, influenced by mitochondria-PM contact sites?
